# Silibinin-mediated metabolic reprogramming attenuates pancreatic cancer-induced cachexia and tumor growth

**DOI:** 10.18632/oncotarget.5843

**Published:** 2015-10-16

**Authors:** Surendra K. Shukla, Aneesha Dasgupta, Kamiya Mehla, Venugopal Gunda, Enza Vernucci, Joshua Souchek, Gennifer Goode, Ryan King, Anusha Mishra, Ibha Rai, Sangeetha Nagarajan, Nina V. Chaika, Fang Yu, Pankaj K. Singh

**Affiliations:** ^1^ The Eppley Institute for Research in Cancer and Allied Diseases, University of Nebraska Medical Center, Omaha, Nebraska 68198, USA; ^2^ Department of Biochemistry and Molecular Biology, University of Nebraska Medical Center, Omaha, Nebraska 68198, USA; ^3^ Department of Biostatistics, University of Nebraska Medical Center, Omaha, Nebraska 68198, USA; ^4^ Department of Pathology and Microbiology, University of Nebraska Medical Center, Omaha, Nebraska 68198, USA; ^5^ Department of Genetics Cell Biology and Anatomy, University of Nebraska Medical Center, Omaha, Nebraska 68198, USA

**Keywords:** pancreatic cancer, cancer metabolism, silibinin, cachexia, c-Myc

## Abstract

Pancreatic ductal adenocarcinoma (PDAC) is the fourth leading cause of cancer-related deaths in the US. Cancer-associated cachexia is present in up to 80% of PDAC patients and is associated with aggressive disease and poor prognosis. In the present studies we evaluated an anti-cancer natural product silibinin for its effectiveness in targeting pancreatic cancer aggressiveness and the cachectic properties of pancreatic cancer cells and tumors. Our results demonstrate that silibinin inhibits pancreatic cancer cell growth in a dose-dependent manner and reduces glycolytic activity of cancer cells. Our LC-MS/MS based metabolomics data demonstrates that silibinin treatment induces global metabolic reprogramming in pancreatic cancer cells. Silibinin treatment diminishes c-MYC expression, a key regulator of cancer metabolism. Furthermore, we observed reduced STAT3 signaling in silibinin-treated cancer cells. Overexpression of constitutively active STAT3 was sufficient to substantially revert the silibinin-induced downregulation of *c-MYC* and the metabolic phenotype. Our *in vivo* investigations demonstrate that silibinin reduces tumor growth and proliferation in an orthotopic mouse model of pancreatic cancer and prevents the loss of body weight and muscle. It also improves physical activity including grip strength and latency to fall in tumor-bearing mice. In conclusion, silibinin-induced metabolic reprogramming diminishes cell growth and cachectic properties of pancreatic cancer cells and animal models.

## INTRODUCTION

Cachexia is a multifactorial syndrome characterized by involuntary weight loss due to skeletal muscle wasting and fat depletion. More than 50% of cancer patients, depending on the type of cancer, suffer from cachexia [1]. Often cancer patients are diagnosed with significant body weight loss that limits therapeutic options. Cachexia in cancer patients leads to weakness, immobility and poor quality of life, which significantly contributes to cancer-related deaths [2]. Cachectic cancer patients exhibit poorer prognosis in comparison to non-cachectic patients. They also have poor survival and response to chemotherapy and radiotherapy as well as significant increase in surgical risk [3]. The etiology of cancer-induced cachexia is not completely understood, but it is considered to be result of a complex interplay of tumor and host factors [4]. The most prominent characteristic of the cachexia is significant skeletal muscle depletion which mainly occurs due to enhanced proteolysis and reduced protein synthesis in myofibers [5]. Considering the role of cancer-induced cachexia in mortality and morbidity of cancer patients, management of cachexia represents a significant unmet medical requirement. Unfortunately, due to the complex nature and limited understanding of the disease, to date there is no established therapeutic regimen for cancer-induced cachexia.

Pancreatic cancer is currently the fourth leading cause of cancer-related death in the United States and is estimated to be second leading cause of cancer-related death in the US by 2030 [6]. Despite improved molecular understanding of disease progression, the five-year survival rate of pancreatic cancer patients is still at 7% [7]. Several factors contribute to the dismal survival of pancreatic cancer patients, including the asymptomatic nature of early stage disease, absence of an effective screening test, and therapeutic resistance [8]. The incidence of cachexia varies widely among different types of cancer. In comparison to other types of cancer, pancreatic cancer has the highest incidence of cachexia; about 80% of patients exhibit cachectic phenotype at the time of diagnosis [9]. Pancreatic cancer-related cachexia significantly affects prognosis of the disease along with reduced postoperative outcome after pancreaticoduodenectomy. It has been recently reported that instead of obesity, cachexia is a key determinant of poor outcome of pancreatic ductal adenocarcinoma patients after surgery [10]. Gemcitabine treatment, the current gold standard therapeutic agent of pancreatic cancer also has been shown to induce cachexia in an experimental model of pancreatic cancer [11], similar to other anti-cancer agents such as taxanes [12]. Considering all these facts, there is an instant need of alternative therapeutic agents that possess anti-cancerous as well as anti-cachectic properties.

Several natural compounds such as graviola and flavonoids have been shown to exhibit tumor metabolism inhibitory [13] and anti-cachectic properties [14]. Silibinin is the major bioactive component of the seed extract of the plant Milk thistle (*Silybum marianum*), which has shown significant anti-proliferative and pro-apoptotic properties in *in vitro* and *in vivo* models of different type of cancers including prostate, colon and renal cell carcinoma [15]. Previous studies have demonstrated that silibinin also exhibits anti-inflammatory properties by regulating the expression of pro-inflammatory cytokines such as IL-6 and IL-8 [16]. Silibinin also suppresses the accumulation of hypoxia inducible factor 1α (HIF1α) and inhibits activity of the mTOR pathway, both of which are important regulators of cancer cell metabolism [17, 18]. Considering all these properties of silibinin, in the present study we have evaluated the anti-cancerous and anti-cachectic role of silibinin in pancreatic cancer by using *in vitro* as well as *in vivo* models. Our results demonstrate that silibinin significantly inhibits the growth of pancreatic cancer cells and induces global metabolic reprogramming. It also suppresses the cachectic potential of pancreatic cancer cells. Our *in vivo* studies demonstrate that silibinin inhibits tumor growth, proliferation and pancreatic cancer-induced cachexia in an orthotopic model of pancreatic cancer. Altogether, our findings demonstrate the anti-cachectic and anti-cancerous activity of silibinin in pancreatic cancer.

## RESULTS

### Silibinin inhibits growth of pancreatic cancer cells

**Figure 1:**
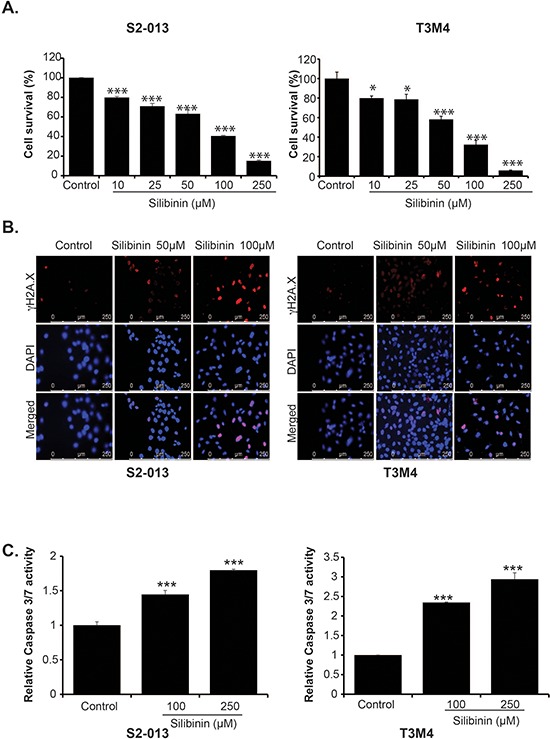
Silibinin inhibits growth of pancreatic cancer cell lines and induces apoptosis **A.** S2-013 and T3M4 cells were treated with different doses of silibinin for 72 h and cell survival was determined by MTT assays. **B.** S2-013 and T3M4 cells were treated with the indicated doses for 48 h and γH2A.X was detected by immunoflourescence assay. **C.** S2-013 and T3M4 cells were treated with different doses of silibinin and Caspase 3/7 activity was determined after 48 h of treatment. Values represented are mean ± SEM. *P ≤ 0.05, **P ≤ 0.01 and ***P ≤ 0.001.

We examined the effect of silibinin on growth of pancreatic cancer cell lines. We evaluated the effect of different doses of silibinin ranging from 10 μM to 250 μM on the survival of S2-013, T3M4, AsPC-1, BxPC-3, MIA PaCa-2 and Panc-1. We observed a dose-dependent inhibition of cell growth in all the cell lines after 72 h treatment (Figure 1A and Supplementary Figure 1A–1D). We further evaluated effect of silibinin on γH2AX levels, a marker for DNA damage and apoptosis, in S2-013 and T3M4 cells using immunofluorescence assay. After 48 h of treatment with 50 μM and 100 μM silibinin, we observed a dose dependent increase in γH2AX level in both S2-013 and T3M4 cells (Figure 1B). Furthermore, we examined the effect of silibinin treatment on Caspase 3/7 activity in S2-013 and T3M4 cells. Our results demonstrate enhanced Caspase 3/7 activity at 48 h post silibinin treatment of S2-013 and T3M4 cells (Figure 1C). Overall, our results demonstrate that silibinin inhibits growth of pancreatic cancer cells in a dose-dependent manner. It also induces DNA damage in pancreatic cancer cells and activates Caspase 3/7-mediated apoptosis.

### Silibinin inhibits cellular metabolism and reduces expression of key metabolic enzymes

**Figure 2:**
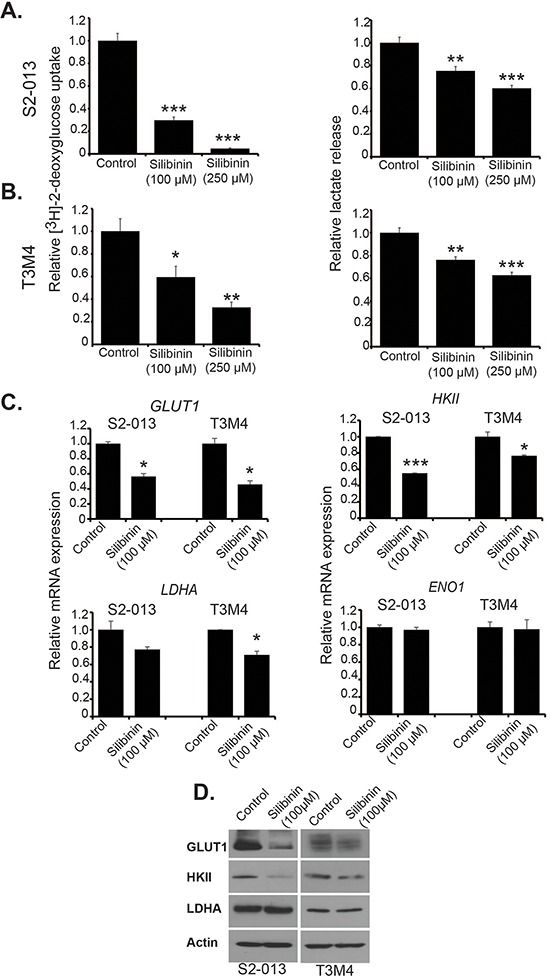
Silibinin inhibits metabolism of pancreatic cancer cells and reduces expression of glycolytic enzymes **A.** S2-013 and **B.** T3M4 cells were treated with different doses of silibinin for 24 h and glucose uptake was determined by performing [^3^H]-2DG uptake assay. Bars represent counts normalized to cell number and plotted relative to controls. Lactate release was determined by colorimetric assay using culture medium of S2-013 and T3M4 cells. Values were normalized to total cell number and represented relative to controls. **C.** S2-013 and T3M4 cells were treated with 100 μM silibinin for 12 h and total RNA was isolated. Relative mRNA levels of *GLUT1*, *HKII*, *LDHA* and *ENO1* were determined by performing qRT-PCR. Actin was used as an internal control. **D.** Protein levels of GLUT1, HKII and LDHA were determined in S2-013 and T3M4 cell lysates from cells treated with solvent control or 100 μM silibinin for 24 h by performing immunoblotting. Actin was used as a loading control. Values shown are mean ± SEM. *P ≤ 0.05, **P ≤ 0.01 and ***P ≤ 0.001.

To explore the effect of silibinin on pancreatic cancer cell metabolism, we investigated glucose uptake and lactate secretion in S2-013 and T3M4 cell lines, 24 h post treatment with 100 μM and 250 μM silibinin. We observed significant decrease in glucose uptake and lactate release in both cell lines in a dose-dependent manner (Figure 2A and 2B). Reduction in lactate release was not as prominent as in case of glucose uptake. It may be due to the contribution of other metabolic pathways such as glutaminolysis in lactate secretion [19]. To determine the mechanistic basis of such metabolic changes, we investigated the effect of silibinin on glycolytic gene expression by performing qRT-PCR. We observed a significant reduction in mRNA expression of *GLUT1, HKII* and *LDHA* after silibinin treatment in S2-013 and T3M4 cells (Figure 2C). We observed no change in mRNA levels of *ENO1* upon silibinin treatment in either cell lines. We also observed reduced GLUT1 and HKII protein expression, but no change in LDHA expression, after silibinin treatment in S2-013 and T3M4 cells (Figure 2D). Thus, our results demonstrate that silibinin inhibits glucose uptake and lactate release in pancreatic cancer cells by down-regulating the expression of key glycolytic enzymes.

### Silibinin induces global metabolic alterations in pancreatic cancer cells

**Figure 3:**
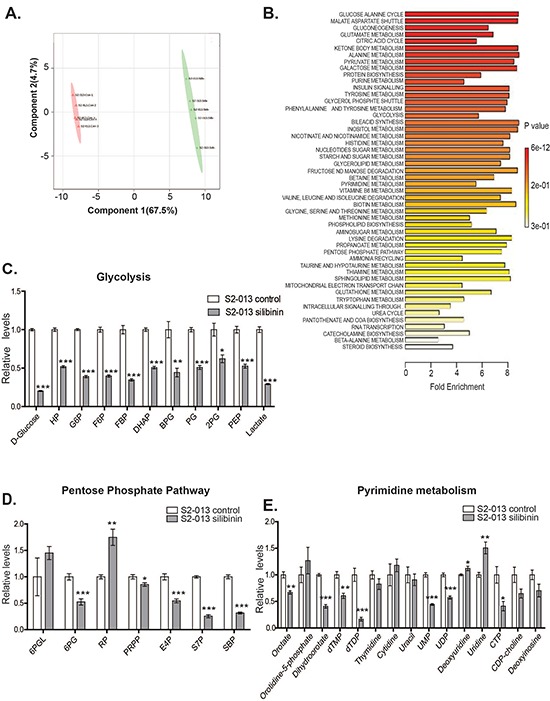
Silibinin modulates glycolysis, PPP and pyrimidine metabolism in pancreatic cancer cells S2-013 cells were treated with solvent control or 100 μM silibinin for 24 h and polar metabolites were extracted and analyzed by performing selected reaction monitoring (SRM) method on a LC-MS/MS mass spectrometer. Peak areas integrated using MultiQuant 2.1 (AB/SCIEX) were normalized to the respective protein concentrations and analyzed by utilizing Metaboanalyst 2.0. Values are represented relative to controls. **A.** PLS-DA (partial least squares discriminant analysis) plot generated from LC-MS/MS data of control and silibinin-treated S2–013 cells metabolites **B.** Pathway enrichment analysis of metabolites from control and silibinin-treated S2-013 cells. **C.** Relative levels of glycolysis pathway metabolites from control and silibinin-treated S2-013 cells. **D.** Relative levels of pentose phosphate pathway metabolites from control and silibinin treated S2-013 cells. **E.** Relative levels of pyrimidine biosynthesis pathway metabolites from control and silibinin-treated S2-013 cells. Values represented are mean ± SEM. *P ≤ 0.05, **P ≤ 0.01 and ***P ≤ 0.001.

Based on our results indicating reduced glycolytic activity in pancreatic cancer cells upon silibinin treatment, we further investigated the effect of silibinin on global metabolic changes in pancreatic cancer cells. We evaluated the polar metabolite profiles of S2-013 cells after silibinin treatment using LC-MS/MS metabolomics. S2-013 cells were treated for 24 h with 100 μM silibinin or vehicle control and then metabolites were extracted followed by sample processing, and data acquisition and analysis. Two dimensional-partial least squares discriminant analysis (2D-PLS-DA) plot of global metabolite content indicated that silibinin-treated S2-013 cells clustered differently from vehicle treated S2-013 cells (Figure 3A). Quantitative enrichment analysis (QEA) of the metabolites demonstrates that glucose-alanine pathway metabolites are highly affected after silibinin treatment (Figure 3B). Furthermore, analysis of individual metabolites revealed that silibinin treatment leads to significant reduction in the levels of several glycolytic intermediate metabolites, including D-glucose (~75%), glucose-6-phosphate, fructose-6-phosphate, fructose-bisphosphate and lactate (~50%) (Figure 3C), suggesting impaired glycolysis in silibinin-treated S2-013 cells. Upholding our previous observation that silibinin treatment reduces glucose uptake and lactate release, our metabolomics data indicated reduced glucose and lactate levels in silibinin-treated S2-013 cells. We also observed significant reduction in pentose phosphate pathway (PPP) metabolites, including 6-phosphogluconate (~50%), erythrose-4-phosphate (~40%), sedoheptulose-7-phosphate and sedoheptulose bis-phosphate (~ 70%) after treatment with silibinin (Figure 3D). The PPP (oxidative arm) mainly originates from the first committed step of glycolysis and is required for the biosynthesis of ribonuleosides, which are the building blocks of nucleic acid synthesis [20]. We observed significant reduction in several nucleotides, including UMP (~50%), CTP (~50%) and UDP (~30%) and their precursors in silibinin-treated S2-013 cells (Figure 3E). Overall, our LC-MS/MS metabolomics-based studies demonstrate that silibinin treatment significantly inhibits glycolytic pathway in pancreatic cancer cells and thus results in reduced pentose phosphate pathway activity and nucleoside synthesis.

### Silibinin inhibits STAT3 signaling and reduces c-MYC expression

**Figure 4:**
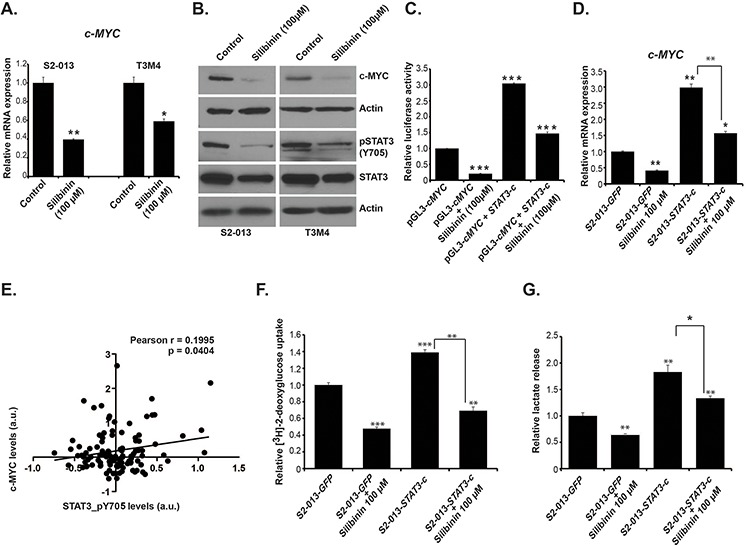
Silibinin reduces expression of c-MYC by inhibiting STAT3 activation **A.** S2-013 and T3M4 cells were treated with 100 μM silibinin for 12 h and total RNA was isolated. Relative mRNA level of *c-MYC* was determined by qRT-PCR. **B.** S2-013 and T3M4 cells were treated with 100 μM silibinin for 24 h and c-MYC protein level was determined by immunoblotting. pSTAT3, and total STAT3 level were determined by immunoblotting using cell lysates of S2–013 and T3M4 cells treated with 100 μM silibinin for 24 h. Beta-actin was utilized as a loading control. **C.** HEK-293T cells were transfected with vector control (GFP) or STAT3-c plasmid and after 36 h of transfection cells were treated with silibinin for 24 h. Relative *c-MYC* promoter activity was determined by performing dual-luciferase assays. The bar charts represent the relative c-Myc activity after normalization to renilla-luciferase activity, as compared to the control. **D.** S2-013 cells were transfected with vector control (GFP) or STAT3-c plasmid and after 36 h of transfection, cells were treated with silibinin for 24 h. Total RNA was isolated and relative mRNA levels of *c-MYC* were determined by performing qRT-PCR. **E.** Pearson correlation analysis of pSTAT3 and c-MYC in 106 human pancreatic cancer patient primary tumor specimen data from TCGA. **F.** S2-013 cells were transfected with vector control (GFP) or STAT3-c plasmid and after 36 h of transfection, cells were treated with silibinin for 24 h. Glucose uptake was determined by performing [^3^H]-2DG uptake assay. Bars represent counts normalized to cell number and plotted relative to controls. **G.** Lactate release was determined by colorimetric assay using culture medium of transfected and silibinin-treated or control cells. *P ≤ 0.05, **P ≤ 0.01 and ***P ≤ 0.001.

Proto-oncogene c-*MYC* is considered as a master regulator of cellular metabolism, growth and proliferation and is deregulated in multiple solid tumors, including pancreatic cancer [21]. Based on our observations of global metabolic alterations in silibinin-treated S2-013 and T3M4 cells, we further evaluated the effect of silibinin on *c-MYC* expression. Our real-time PCR analyses demonstrated reduced expression of *c-MYC* gene in S2-013 and T3M4 cells (Figure 4A). Furthermore, we observed reduced protein levels of c-MYC in S2-013 and T3M4 cells upon silibinin treatment, as determined by western blotting (Figure 4B). It has been reported that silibinin exhibits its anti-cancerous effect through modulation of several pro-inflammatory and growth signaling pathways [22]. In order to understand the molecular basis of silibinin-mediated c-MYC expression regulation and metabolic reprogramming of pancreatic cancer cells, we investigated the effect of silibinin treatment on STAT3 activation, which enhances *c-MYC* expression [23]. We observed a reduction in STAT3 levels and even stronger reduction in pSTAT3 (pY705) levels in S2–013 and T3M4 cells upon silibinin treatment (Figure 4B). To investigate the direct transcriptional regulation of *c-MYC* expression by STAT3 activation under silibinin treatment, we investigated the effect of constitutively active STAT3 on *c-MYC* promoter activity. Transient transfection of HEK-293T cells with a plasmid construct expressing constitutively active STAT3, along with pGL3-*c-MYC* reporter plasmid indicated over three-fold increase in *c*-*MYC* promoter activity, which could be significantly diminished by silibinin treatment (Figure 4C). Next, we evaluated if constitutively active STAT3 upregulates *c-MYC* mRNA levels in S2-013 cells, with and without silibinin treatment. We observed increased *c-MYC* expression in constitutively active STAT3 transfected S2–013 cells and the upregulation was significantly abrogated by silibinin treatment (Figure 4D). To determine the causal relationship between STAT3 activation and c-MYC expression in human pancreatic cancer, we investigated the TCGA pancreatic cancer dataset for a correlation between pSTAT3 levels and c-MYC protein expression in 106 primary tumor specimens. We observed a modest but statistically significant correlation between protein levels of pSTAT3 and c-MYC (Figure 4E). Furthermore, exogenous expression of constitutively active STAT3 also increased glucose uptake and lactate secretion in S2-013 cells that was significantly reduced upon silibinin treatment (Figure 4F–4G). Thus, our results demonstrate that silibinin diminishes *c-MYC* expression by inhibiting STAT3 activation.

### Silibinin inhibits tumor growth in orthotopic implantation model of pancreatic cancer

**Figure 5:**
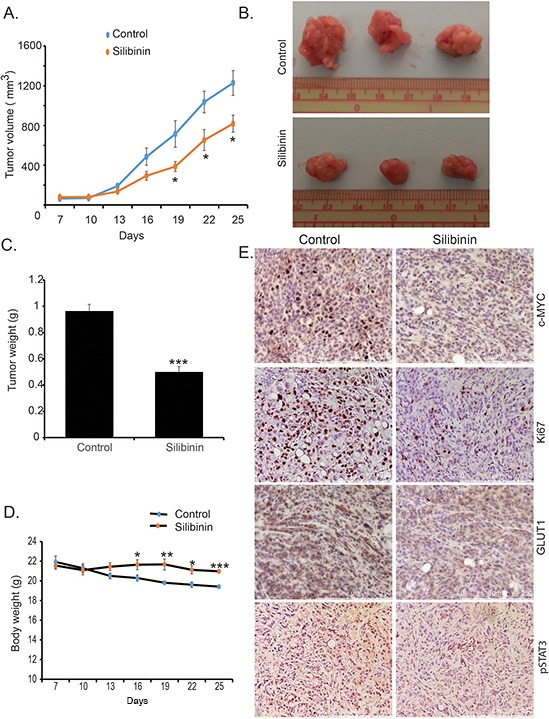
Silibinin inhibits pancreatic tumor growth in orthotopic implantation model S2-013 cells were orthotopically implanted into the pancreas of female athymic nude mice. After seven days of implantation, mice were treated with silibinin or vehicle control. After three weeks of treatment, mice were sacrificed. **A.** Tumor volumes are plotted over indicated periods. **B.** Representative images of tumor upon necropsy. **C.** Bar chart represents average tumor weight upon necropsy. **D.** Body weight of mice during the period of treatment measured by weighing each third day. **E.** Expression of c-MYC, Ki-67, GLUT1, and pSTAT3 in tumor tissue as determined by immunohistochemistry. Values shown are mean ± SEM. *P < 0.05, **P ≤ 0.01, and ***P ≤ 0.001.

To investigate the anti-cancerous effect of silibinin in *in vivo* conditions, we utilized an orthotopic implantation mouse model of pancreatic cancer. We implanted 0.5 × 10^6^ S2-013 cells into the pancreas of 6–8 week old female athymic nude mice. After seven days of implantation, mice were randomly divided into two groups for control and silibinin treatment. After three weeks of daily treatment with silibinin, mice were sacrificed and carcass weight, tumor weight, tumor volume and muscle weight were recorded. We observed a significant reduction in tumor growth rate and tumor volume upon necropsy in silibinin-treated tumor-bearing mice in comparison to the control group (Figure 5A–5C). We also observed a significant difference between body weight of the control group and silibinin-treated mice (Figure 5D), with significantly reduced weight loss in the silibinin-treated tumor-bearing mice. Furthermore, we observed reduced tumor cell proliferation in the silibinin-treated group in comparison to the control group, as evident by reduced Ki67-positive cells in the silibinin-treated tumor sections (Figure 5E). We also observed reduced tumoral c-MYC, GLUT1, and pSTAT3 expression in silibinin-treated mice in comparison to the controls (Figure 5E), which corroborates our results from cell culture-based studies. Furthermore, we evaluated the blood glucose level and glucose tolerance in healthy controls, tumor-bearing mice and tumor-bearing mice treated with silibinin. We observed reduced blood glucose levels and improved glucose tolerance in silibinin-treated tumor-bearing mice in comparison to the control tumor-bearing mice (Supplementary Figure 2A and 2B). Overall, we demonstrate that silibinin treatment leads to reduced tumor growth and proliferation in S2-013 tumor-bearing mice. Furthermore, silibinin treatment reduces STAT3 activation, c-MYC and GLUT1 expression, and the number of proliferating cells in pancreatic tumors, while preventing tumor-induced body-weight loss.

### Silibinin attenuates pancreatic cancer-induced cachexia *in vitro* as well as *in vivo*

**Figure 6:**
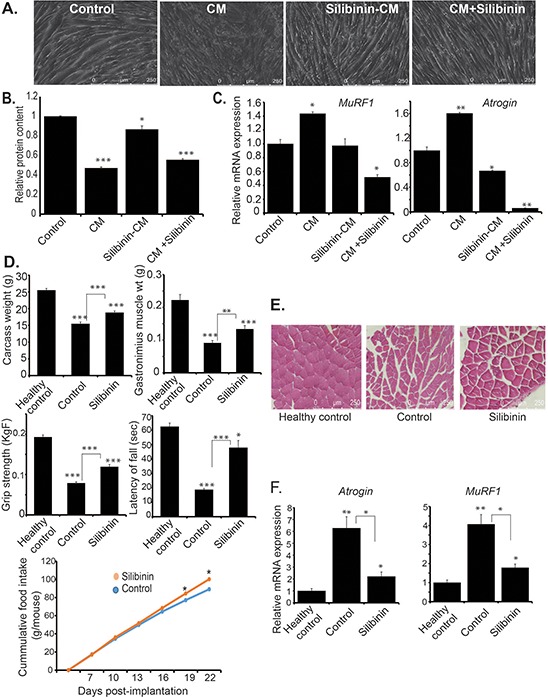
Silibinin reduces cachectic potential of pancreatic cancer **A.** Differentiated myotubes from C2C12 cells were cultured in control, S2-013-conditioned medium, silibinin-pretreated S2-013-conditioned medium, and S2-013-conditioned medium with silibinin for 72 h and bright-field images were represented for individual treatments. **B.** Total protein content from C2C12 myotubes cultured in different conditions was determined by Bradford assay and relative level of protein is represented in the bar graph. **C.** Differentiated myotubes from C2C12 cells were cultured in control, S2-013-conditioned medium, silibinin-pretreated S2-013-conditioned medium, S2-013-conditioned medium with silibinin for 24 h and total RNA was isolated. Relative gene expression of *MuRF1* and *Atrogin* was determined by performing qRT-PCR. Beta-actin was used as a loading control. **D.** Carcass weight upon necropsy, gastrocnemius muscle weight upon necropsy, grip strength, latency of fall, and cumulative food intake over the course of treatment for healthy controls (non-tumor-bearing), control tumor-bearing and silibinin-treated tumor-bearing mice at the time of sacrifice. Grip strength and latency of fall was determined after 23 days of implantation, using grip strength meter and rotarod test. **E.** H & E staining of gastrocnemius muscle sections from healthy controls, control tumor-bearing and silibinin-treated tumor-bearing mice. **F.** Total RNA was isolated from gastrocnemius muscle specimens of healthy controls, control tumor-bearing and silibinin-treated tumor-bearing mice. Relative mRNA levels of *MuRF1* and *Atrogin* were determined by performing qRT-PCR. Values shown are mean ± SEM. *P < 0.05, **P ≤ 0.01, and ***P ≤ 0.001.

Previously we have reported that diet-induced metabolic reprogramming inhibits pancreatic cancer-induced cachexia [24]. Based on our observations of reduced body weight loss in silibinin-treated tumor-bearing mice and metabolic alterations in silibinin-treated pancreatic cancer cells, we further evaluated the role of silibinin in preventing cancer-induced cachexia. Increased muscle atrophy is a key feature of cancer-induced cachexia. Hence, we evaluated the effect of silibinin treatment on cancer cell-conditioned medium-induced C2C12 myotube atrophy. We observed reduced myofiber degradation and proteolysis upon treatment with cancer cell-conditioned medium that was derived from silibinin pre-treated S2-013 cancer cell (Figure 6A–6B). We also observed similar protection with direct treatment of myotubes with silibinin (Figure 6A–6B). Furthermore, we evaluated the expression of muscle atrophy-associated genes *MuRF1* and *Atrogin* in conditioned medium-treated C2C12 myotubes. We observed reduced expression of both *MuRF1* and *Atrogin* in myotubes treated with silibinin-pretreated S2-013 cell-derived conditioned medium as well as in myotubes directly treated with silibinin along with the S2-013 cell-conditioned medium, in comparison to the myotubes treated with S2-013 cell-conditioned medium alone (Figure 6C). Furthermore, we evaluated the effect of silibinin treatment on the cachectic phenotype in S2-013 pancreatic cancer cell-implanted mice. We observed significant increase in carcass weight, muscle weight, grip strength and latency of fall in silibinin-treated tumor-bearing mice in comparison to the solvent control-treated tumor-bearing mice (Figure 6D). We also observed improved muscle fiber morphology and reduced fibrosis in muscle tissues from silibinin-treated tumor-bearing mice in comparison to controls (Figure 6E). Further, we evaluated *MuRF1* and *Atrogin* expression in muscles of healthy control, S2-013 tumor-bearing control mice and silibinin-treated S2-013 tumor-bearing mice. We observed reduced expression of mRNA levels of *MuRF1* and *Atrogin* in silibinin-treated tumor-bearing mice in comparison to controls (Figure 6F). We also evaluated the effect of silibinin on gene expression of pro-inflammatory cytokines in pancreatic cancer cells as well as controls and silibinin-treated tumor tissues. We observed reduced expression of *TNF-alpha* and *IL-6* (Supplementary Figure 3A–3D). Thus, our results demonstrate that silibinin treatment attenuates pancreatic cancer-induced cachexia in orthotopic implantation models of pancreatic cancer.

## DISCUSSION

The anti-cancerous property of plant-derived flavanoid silibinin has been shown in several types of cancer, but its effect on the metabolic phenotype of cancer cells and cancer-cachexia, a systemic metabolic disorder induced by the metabolic needs of a growing tumor, has not been studied. In the current studies, we explored the effect of silibinin on pancreatic cancer cell growth, metabolic alterations, and cachectic potential using *in vitro* as well as *in vivo* models. Altered metabolism is considered a hallmark of cancer cells which contributes to maintain uncontrolled growth and proliferation by providing sufficient biomass and energy [25]. Therapeutic approaches to combat cancer have been focused on targeting the rapid proliferation of cancer cells. Since altered metabolism of transformed cells significantly contributes to cellular proliferation, targeting metabolic pathways of cancer cells shows promise in the area of cancer therapeutics [26]. Cancer cells display an altered metabolic phenotype in order to fulfill their enhanced energy and biomass synthesis requirements for uncontrolled growth [27]. A key metabolic alteration exhibited by most cancer cells is enhanced aerobic glycolysis, a phenomenon known as “the Warburg effect” which provides several metabolic benefits to proliferating cancer cells [28]. In addition to enhanced glycolytic activity, cancer cells also exhibit addiction to glutamine, which plays a very important role in bioenergetics and biosynthesis processes of cancer cells [29]. Recently, several studies have demonstrated the therapeutic efficacy of metabolic inhibitors in different types of cancer. Glycolytic inhibitors 2-Deoxyglucose, 3-Bromopyruvate have shown anti-cancerous property in different preclinical models of cancer [30]. Several studies have reported the altered metabolic phenotype of pancreatic cancer cells, governed by genetic alterations such as *K-RAS* mutations and growth signaling pathways [31].

Our current study demonstrates that silibinin exhibits its anti-proliferative action through modulation of pancreatic cancer cell metabolism, and by inducing apoptosis. We observed reduced glucose and lactate secretion after treatment of cancer cells with silibinin. Glucose is one of the primary sources of cellular energy and biomass production [32], so inhibition of key metabolic drivers that regulate glucose metabolism by silibinin would inhibit the cellular growth and proliferation. Aberrantly increased expression of glucose transporters such as GLUT1 plays a significant role in aggressiveness of multiple cancers, including pancreatic cancer [33]. Recently, our lab has demonstrated that GLUT1 expression is regulated by mucin family protein MUC1 in a hypoxia-dependent manner and increased glucose uptake by tumors is correlated to an aggressive phenotype [34, 35]. We also observed reduced expression of GLUT1 after silibinin treatment in pancreatic cancer cells. As expected, the reduced expression of GLUT1 also resulted in reduced glucose uptake by silibinin-treated cells. GLUT1 expression was also reduced in silibinin-treated tumor tissues in orthotopic model of pancreatic cancer. Along with GLUT1, we also observed reduced expression of HKII, a key glycolytic enzyme that is critical for tumor initiation and maintenance [36]. Thus, inhibition of GLUT1 and HKII expression by silibinin may be responsible for the reduced glucose uptake and glycolytic activity in silibinin-treated pancreatic cancer cells.

Our mass spectrometry-based metabolomics studies demonstrate that silibinin treatment leads to alterations in the global metabolite profile of pancreatic cancer cells. Glucose has several catabolic and biosynthetic fates in proliferating cancer cells [37], hence, the altered global metabolite profile of pancreatic cancer cells revealed altered glucose metabolism after silibinin treatment. Metabolic alterations in cancer cells by natural compounds like, flavonoids have been reported in several studies. Plant-derived bioactive compounds like quercetin, genistein, naringenin, and catechin have also been shown to alter glucose uptake in cancer cells [38]. We observed significant down regulation of several glycolytic intermediates after silibinin treatment, further corroborating our findings of silibinin-mediated reduction in glucose uptake, lactate secretion, and glycolytic enzymes expression.

Enhanced glycolytic metabolism directly contributes to proliferation of cancer cells, specifically providing higher levels of glycolytic intermediates needed for biomass synthesis and several anabolic reactions [39]. The glycolytic intermediate, glucose-6-phosphate, serves as substrate for pentose phosphate pathway, which provides several metabolic intermediates needed for cellular growth and proliferation and has been reported to be deregulated in several types of cancer [20]. We observed reduced levels of pentose phosphate pathway metabolites in silibinin-treated pancreatic cancer cells, which may be due to reduced glucose uptake by these cells. Every proliferating cell requires replication of its fundamental components, including DNA, RNA, proteins, and lipids. The pentose phosphate pathway is the major source of ribonucleotide synthesis and NADPH, which are crucial for nucleotide and lipid biosynthesis, respectively [20]. We also observed reduced pyrimidine biosynthesis pathway metabolites in silibinin-treated cells, indicating impaired nucleoside biosynthesis in silibinin-treated cells. Overall, our mass spectrometry-based metabolomics data support cell-based physiological assays indicating impaired glucose metabolism leading to global metabolic alterations in pancreatic cancer cells in response to silibinin treatment. As we observed reduced levels of key metabolites important for the bioenergetic and biosynthesis processes of cancer cells, metabolic reprogramming may be the fundamental aspect of silibinin-mediated inhibition of cancer cell growth and proliferation.

Studies from several laboratories have demonstrated c-MYC as an important regulator of growth, proliferation and metabolism of cells [21]. c-MYC expression is essential for several metabolic alterations of cancer cells in order to fulfill their bioenergetic and biosynthetic demands. It regulates glycolysis, nucleotide synthesis and protein synthesis, which are basic requirements of proliferating cancer cells [40]. We observed reduced expression of *c-MYC* in silibinin-treated cells. Transcription factor STAT3 is a positive regulator of *c-MYC* expression and is essential for cellular transformation [23]. It has been reported that silibinin inhibits STAT3 activation in several preclinical cancer models [41]. STAT3 also plays a very critical role in metabolic reprogramming of cancer cells by driving metabolism towards aerobic glycolysis by transcriptional modulation [42]. Our results also demonstrate that treatment of pancreatic cancer cells with silibinin leads to reduced phosphorylation of STAT3. We have also demonstrated that STAT3-mediated up regulation of *c-MYC* transcription is compromised after silibinin treatment. Therefore, our study provides strong support of STAT3 mediated *c-MYC* regulation and its inhibition by silibinin. However, the exact role of *c-MYC* in cachexia is not fully defined and will be evaluated by future studies.

Silibinin inhibits growth and proliferation of breast cancer, prostate cancer and colon cancer cells by different mechanisms, including reduced DNA synthesis, cell cycle arrest and modulation of growth signaling pathways [22]. We also observed reduced tumor growth and proliferation of pancreatic tumors in an orthotopic model of pancreatic cancer. A reduction in c-MYC and GLUT1 expression in tumors treated with silibinin further validates cell culture-based assays. Most pancreatic cancer patients (~70%) suffer from glucose intolerance, which further worsens over the pathogenesis of the disease [43]. We observed improved glucose tolerance and reduced blood glucose levels in tumor-bearing mice treated with silibinin.

About 80% of pancreatic cancer patients suffer from cachexia, which significantly contributes to mortality and morbidity of the disease [44]. Cachexia is a complex disorder that results from tumor-host interplay and metabolic alterations causing increased energy expenditure, enhanced catabolism in liver, increased fat oxidation and proteolysis in muscle [45]. We have shown earlier that metabolic reprogramming of cancer cells by dietary intervention can inhibit the cachectic phenotype of pancreatic cancer [24]. Along with metabolic reprogramming of pancreatic cancer cells, we also observed anti-cachectic properties of silibinin in *in vitro* as well as *in vivo* models. We observed significant improvement in body weight of tumor-bearing mice treated with silibinin as well as reduced myotube degradation induced by cancer cell-conditioned medium pretreated with silibinin. Previous studies have demonstrated that silibinin exhibits anti-inflammatory properties by reducing secretion of several pro-inflammatory cytokines [46]. Silibinin also exhibits its anti-inflammatory properties by reducing NF-κB activity and inhibiting the expression of several cytokines, including IL-1β, IL-6, IL-10, IL-12, and TNF-α [47]. We also observed reduced expression of *TNF-alpha* and *IL-6* in silibinin-treated cancer cells as well as in tumor tissues from tumor-bearing mice treated with silibinin. On the basis of our results, we conclude that anti-cachectic effect of silibinin might be mediated through metabolic reprogramming as well as suppression of pro-inflammatory cytokines.

In conclusion, we have demonstrated that bioactive compound silibinin exhibits anti-cancerous and anti-cachectic properties in *in vitro* as well as *in vivo* models of pancreatic cancer. It induces metabolic reprogramming in pancreatic cancer cells by reducing expression of a key metabolic regulator, c-MYC, through reduced activation of STAT3. Furthermore, we observed improved glucose homeostasis and diminished cachexia in tumor-bearing mice treated with silibinin.

## MATERIALS AND METHODS

### Cell culture and reagents

Human pancreatic cancer cell lines S2-013 (pancreatic tumor cell line SUIT-2 derived from a liver metastasis), T3M4, PANC-1, BxPC-3 AsPC-1 and MIA PaCa-2 from ATCC were cultured in DMEM medium supplemented with 5% fetal bovine serum, 100 I.U./ml penicillin, 100 μg/ml streptomycin, and incubated at 37°C in a humidified incubator with 5% CO_2_, as described previously [48]. C2C12 mouse myoblasts were cultured in DMEM with 5% FBS and differentiated to myotubes as described previously [24]. Silibinin, 3-[4, 5-dimethylthiazol-2-yl]-2, 5-diphenyltetrazolium bromide (MTT) were purchased from Sigma-Aldrich, St. Louis, MO, USA.

### Cell viability, growth and Caspase 3/7 assay

Cell viability was determined by MTT assay as described previously [24]. Caspase 3/7 activity was determined by Promega Caspase-Glo kit (Madison, WI, USA) as described previously [24].

### Glucose uptake and lactate release assay

Glucose uptake assay was performed by using tritiated 2-deoxy-D-glucose as described previously [24]. The experiments were performed in triplicate and results were normalized to total cell number. Lactate release in medium was determined by Lactate Assay Kit (Eton Bioscience Inc., San Diego, CA, USA), as per the manufacturer's protocol.

### RNA isolation and qRT-PCR

Total RNA from cells or tissue lysate was extracted by using TRIzol reagent (Invitrogen, Carlsbad, USA) as per manufacturer's protocol. Complementary DNA (cDNA) was synthesized using Verso-cDNA synthesis kit (Thermo scientific, MA, USA) following manufacturer's protocol. Expression level of genes was measured by qRT-PCR using SYBR Green master mix (Applied Biosystems, NY, USA) and an ABI 7500 thermo cycler. Reaction mix was prepared with 3 μl cDNA, 2 μl primers and 5 μl SYBR Green master mix as mentioned previously [24]. PCR conditions were used: 95°C for 5 min and 95°C for 10s, 60°C for 60s (40 cycles). Beta-actin was utilized as an internal control. Relative expression levels of genes were determined by the ΔΔCt method [24].

### Immunoblotting

Protein isolation and western blotting were performed as described previously [49]. Briefly, cells were washed twice with PBS and lysed in radioimmuno precipitation assay (RIPA) lysis buffer by incubating at 4°C on a rotatory shaker for 30 min. To remove the cell debris, lysates were centrifuged at 13000 rpm for 10 min. Protein concentration was measured by Bradford assay. Equal amount of protein was used for western blotting. Primary antibodies against GLUT1 (Abcam, Cambridge, UK), c-Myc (clone 9E10; Santa Cruz Biotechnology, Dallas, Texas, USA), HKII, LDHA, STAT3, pSTAT3, anti-phospho histone H2A. X (Cell Signaling Technology, Danvers, MA, USA) and actin (Developmental Studies Hybridoma Bank, Iowa City, IA) were utilized for probing specific proteins.

### Transfection of plasmids

Plasmids were transfected as described previously [50]. EF.STAT3C.Ubc.GFP, vector control, c-MYC promoter-luciferase reporter and renilla-luciferase constructs (Addgene) were transfected by using X-treme GENE 9 transfection reagent (Roche Diagnostics, Indianapolis, Indiana, USA) as per the manufacturer's protocol.

### Luciferase assay

Luciferase assay was performed as described previously [24]. c-*MYC*-promoter (del1)-luciferase reporter construct and *STAT3-c*.Ubc.GFP plasmid (constitutively active STAT3; STAT3-c) were purchased from Addgene (Cambridge, MA, USA). Cells were transfected with 250 ng reporter and 750 ng activator/vector plasmid and 36 h post transfection, cells were treated with silibinin. A synthetic *Renilla* luciferase reporter pRL-TK was utilized as a transfection control. Luciferase activity was determined by utilizing Dual-Luciferase Reporter Assay System (Promega, WI, USA) as per manufacturer's instructions.

### Immunofluorescence staining

S2-013 and T3M4 cells were seeded at ~ 40% density on sterile glass cover slips in 12 well plates. The next day cells were treated with indicated amount of silibinin for 48 h. After treatment cells were washed with PBS to remove the media and immunofluorescence staining was performed as described earlier [51] using phosphorylated H2A.X (γH2A.X) antibody (1:500 dilution). Images were captured at 20X magnification using an inverted microscope (DMI600B from Leica) and processed by using Leica LAS AF software.

### Immunohistochemistry

Immunohistochemistry was performed as described previously [35]. Tumor sections prepared from control and silibinin-treated groups were stained with Ki67 (Thermo Fisher Scientific, Waltham, MA, USA) and c-Myc (clone 9E10; Santa Cruz Biotechnology, Dallas, Texas, USA), pSTAT3 (Cell Signaling Technology, Danvers, MA, USA) and GLUT1 (Abcam, Cambridge, UK) primary antibodies. Images were captured at 20X magnification using an inverted microscope (Leica, DMI600B) and processed by using Leica LAS AF software.

### Animals and orthotopic implantation

All the animal experiments performed in this study were approved by University of Nebraska Medical Center institutional animal care and use committee (IACUC). Athymic female nude mice (NCr-nu/nu) were inhouse bred and 6–8 week old mice were used for orthotopic implantation. 0.5 × 10^6^ S2-013 cells were injected into the pancreas of each female athymic nude mouse and 7 days post-implantation mice were divided in groups of eight animals each. Eight age and sex matched mice, without any tumor cell injection, were utilized as healthy controls. Animals were treated with 200 mg/kg silibinin or solvent control daily. Tumor volume, food consumption and body weight were recorded regularly. After 18 days of treatment, all the mice were euthanized and tumor weight, tumor volume, muscle weight, carcass weight, etc. were measured. Tumor tissue, liver, spleen and muscles were flash frozen in liquid nitrogen and formalin fixed for further analysis.

### Measurement of grip strength and rotarod test

We utilized a grip strength meter (Columbus Instruments, OH, USA) to assess grip strength. On the 16^th^ day of treatment, we acclimatized mice to the procedure room for 15 minutes and measured grip strength as per manufacturer's instructions. We recorded the peak force produced by mouse grip and utilized the average value of three measurements within 2 minutes for analysis. We evaluated coordination and body mobility of mice by rotarod test (Rotamex-5, Columbus Instruments, OH, USA). After 15 minutes of acclimatization in the procedure room, we placed mice on rotarod, which was rotating at a speed of 3 rpm. The speed was increased regularly by 1 rpm per 10 seconds. Latency to fall on paper-cushion was recorded. The test was repeated twice after 10-minute intervals. The average time of all the three trials was calculated.

### Metabolite extraction and MS sample preparation

Metabolite extraction was performed as described previously [52]. About 70% confluent S2-013 cells were treated with silibinin for 24 h. After treatment, cells were washed twice with cold PBS and polar metabolites were then extracted with cryogenically cold 80% methanol/water mixture. LC-MS grade water (Sigma, MO, USA) and LC-MS grade methanol (Fischer Scientific, PA, USA) were used. Methanol extracted samples were dried by using a speed vacuum evaporator (Savant Speed Vac^®^ Plus, Thermo Electron Corporation, USA) to evaporate the methanol and lyophilized by using a freeze dry system (Labconco, Kansas City, USA) to remove the water.

### LC-MS/MS experiment and analysis

Lyophilized samples were dissolved in equal amounts of LC-MS grade water and applied to LC-MS/MS analysis, using a multiple reaction monitoring (MRM) method by utilizing AB SCIEX 5500 QTRAP, as described previously [53]. Data acquisition was performed by using Analyst^TM^1.6 software (AB SCIEX) and peaks were integrated by using Multiquant^TM^ (AB SCIEX). Peak areas were normalized by using respective protein concentrations and the resultant peak areas were subjected to relative quantification analyses with MetaboAnalyst 2.0 [54].

### C2C12 differentiation and conditioned medium preparation

C2C12 cells were differentiated to myotubes as described previously [24]. Briefly, C2C12 mouse myoblast cells were cultured in DMEM with 5% FBS. Myoblasts were differentiated by using DMEM with 2% horse serum and 10 μg/ml insulin for 72 h. Differentiation medium was changed after every 24 h. For the preparation of conditioned medium, S2-013 cells were seeded at a density of 50,000 cells/cm^2^. After 12 h of seeding, cells were washed twice with PBS and cultured in serum free medium for the next 24 h. After 24 h, medium was collected and centrifuged at 3000 rpm for ten minutes to remove cell debris. The conditioned media thus collected was then utilized to treat differentiated myotubes or stored at −80°C.

### Measurement of blood glucose and intraperitoneal glucose tolerance test (IPGTT)

Blood glucose level of the mice was measured by using Nova Max Glucose Test Strips (Nova Diabetes Care, Inc., MA, USA) post 16 h starvation. To perform IPGTT, mice were fasted for 16 and then administered 2 g/kg glucose and blood glucose level was measured at different time intervals (0, 30, 60 and 120 minutes after glucose administration) by using Nova Max Glucose Test Strips.

### Statistical analysis

We performed ANOVA (one-way; graph Pad Prism version 4.03) to analyze the treatment responses. We performed Tukey's post-hoc analysis for pair-wise comparisons. We utilized Student's *t*-test for comparisons between two groups. A *p* value of less than 0.05 was considered significant.

## SUPPLEMENTARY MATERIALS FIGURES


