# Supplementary material for: Silibinin-mediated metabolic reprogramming attenuates pancreatic cancer-induced cachexia and tumor growth
Source: Oncotarget. 2015 Oct 16;6(38):41146–61. doi: 10.18632/oncotarget.5843 (PMC4747396; doi:10.18632/oncotarget.5843)
Supplement: Supplementary file 1 [file oncotarget-06-41146-s001.pdf]

SUPPLEMENTARY FIGURES

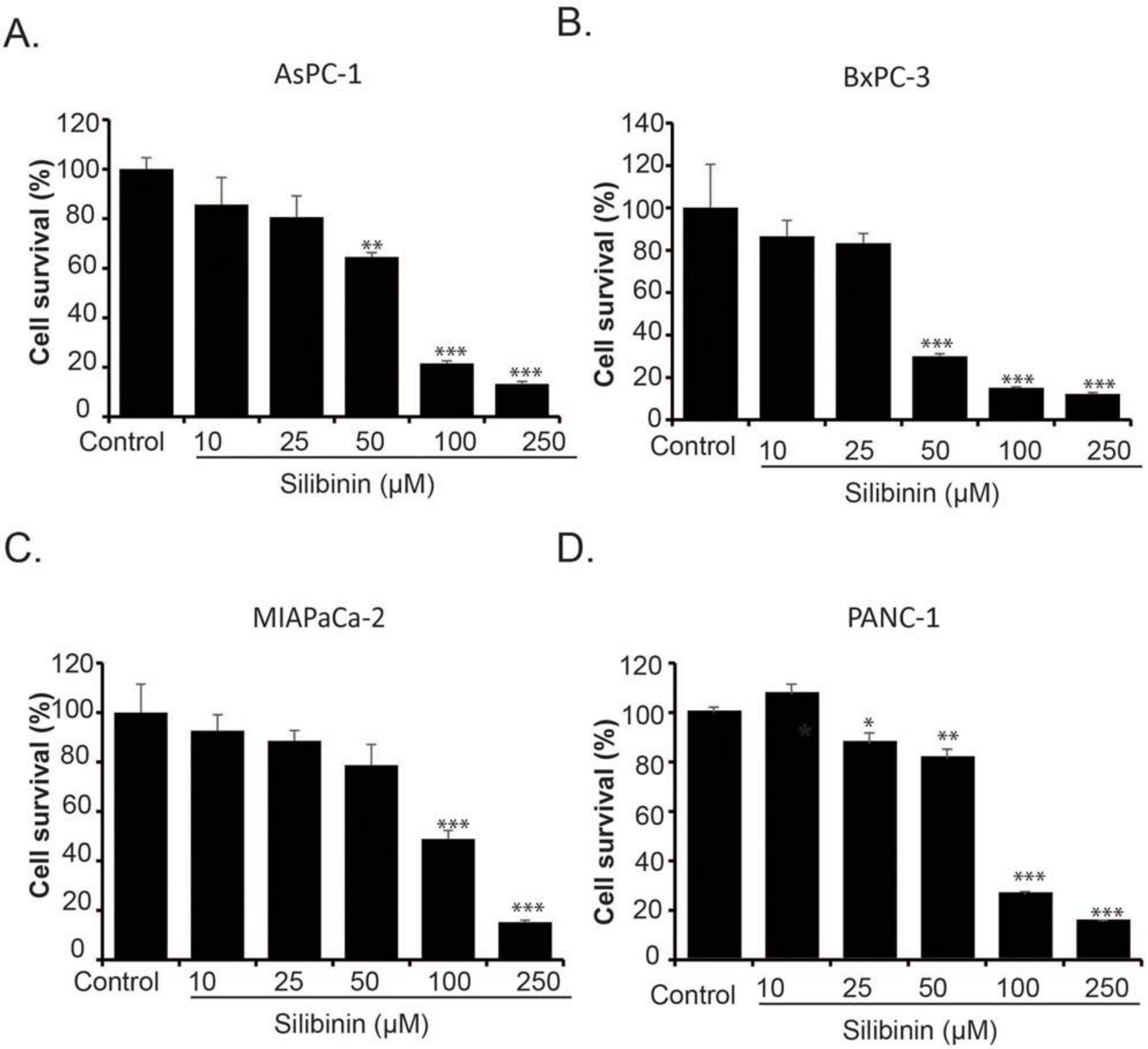

Supplementary Figure S1: Silibinin inhibits growth of pancreatic cancer cell lines and induces apoptosis. A. AsPC-1 **B.** BxPC-3 **C.** MIAPaCa-2 and PANC-1 cells were treated with different doses of silibinin for 72 h and cell survival was determined by MTT assay. *$P \leq 0.05$, **$P \leq 0.01$ and ***$P \leq 0.001$.

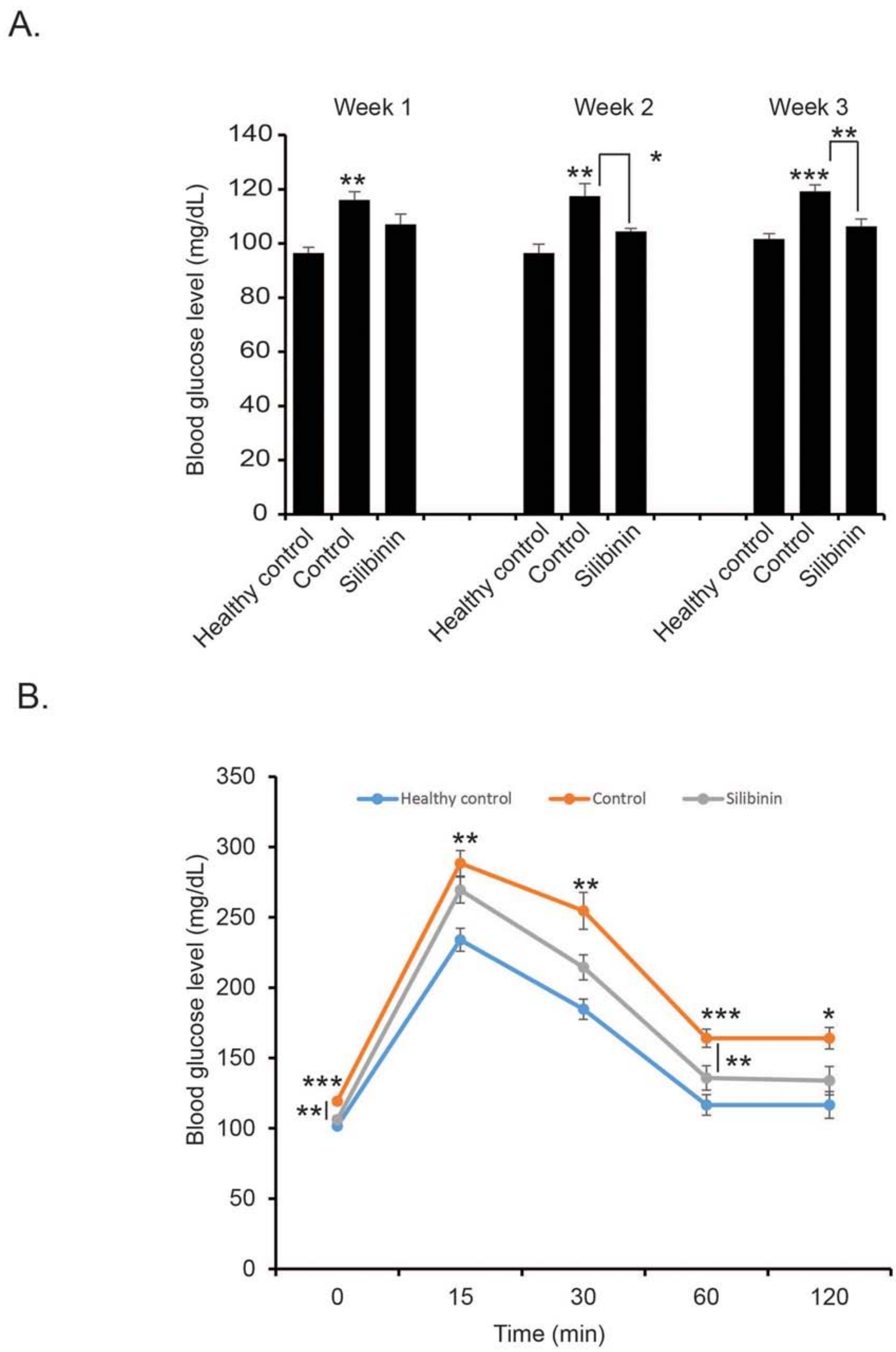

Supplementary Figure S2: Silibinin reduces blood glucose level and improves glucose tolerance. A. Blood glucose level of mice were measured after overnight fasting by using glucose test strips and glucometer. **B.** Intraperitoneal glucose tolerance tests in mice were performed as described in methods section. Representative average glucose levels at different time points are plotted. $*P \leq 0.05$, $**P \leq 0.01$ and $***P \leq 0.001$.

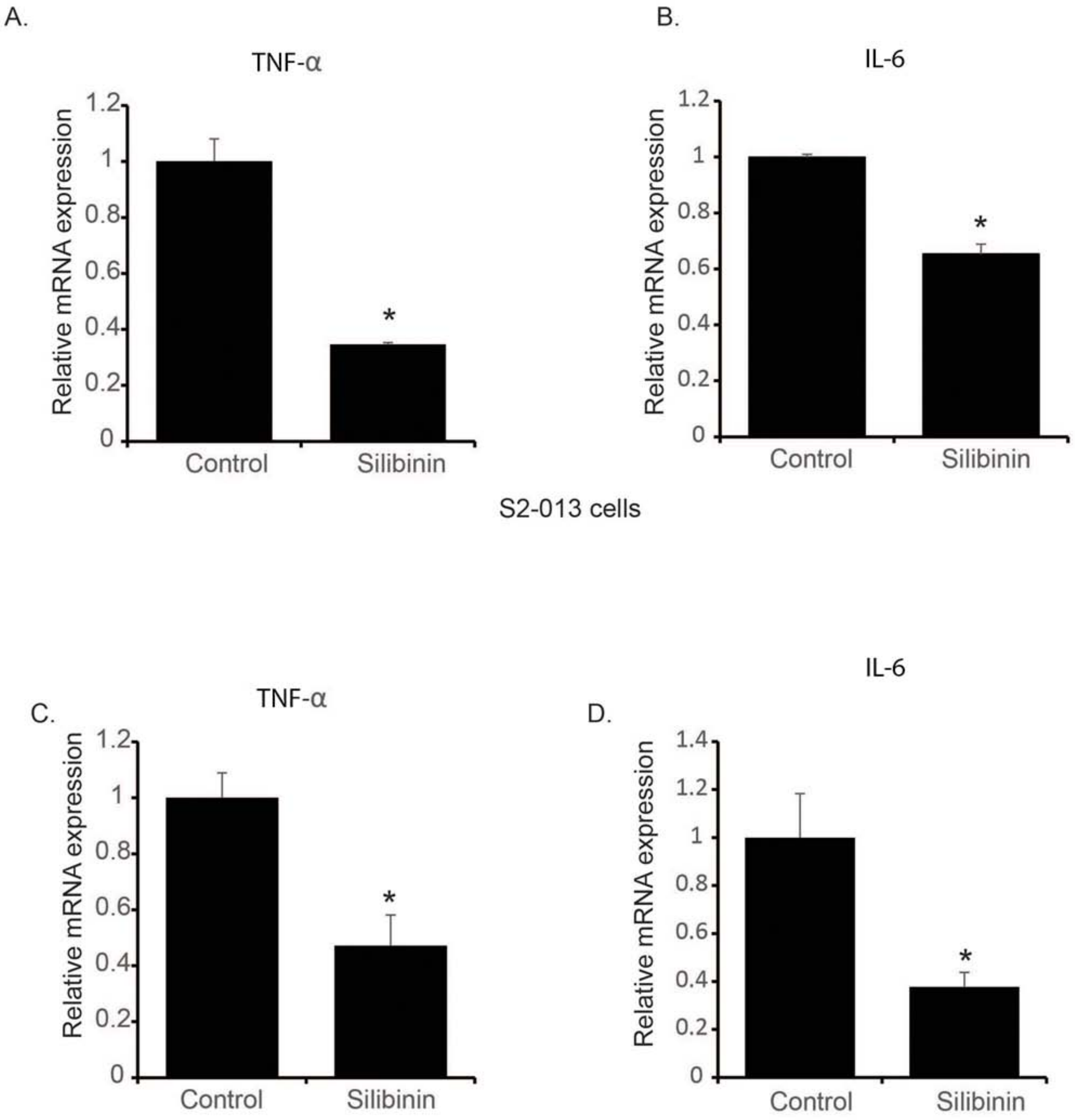

Supplementary Figure S3: Silibinin inhibits *TNF-α* and *IL-6* expression. S2-013 cells were treated with 100 µM of silibinin for 12 h and total RNA was isolated. Relative mRNA levels of **A.** *TNF-α* and **B.** *IL-6* were determined by performing qRT-PCR. Actin was used as an internal control. Total RNA was isolated from primary tumors from control and silibinin-treated mice and relative mRNA levels of **C.** *TNF-α* and **D.** *IL-6* were determined by performing qRT-PCR. Actin was utilized as internal control. Values represented are mean ± SEM. $^*P < 0.05$.
